# Acute febrile encephalopathy in adults from Northwest India

**DOI:** 10.4103/0974-2700.66520

**Published:** 2010

**Authors:** Ashish Bhalla, Vika Suri, Subhash Varma, Navneet Sharma, Sushil Mahi, Paramjeet Singh, Niranjan K Khandelwal

**Affiliations:** Department of Internal Medicine, Postgraduate Institute of Medical Education and Research, Chandigarh, India; 1 Department of Radiodiagnosis, Postgraduate Institute of Medical Education and Research, Chandigarh, India

**Keywords:** Altered mentation, encephalopathy, fever, meningoencephalitis, tropics

## Abstract

**Background:**

Acute onset fever with altered mentation is a common problem encountered by the physician practicing in tropical countries. Central nervous system (CNS) infections are the most common cause resulting in fever with altered mentation in children.

**Aim:**

In this study, we have tried to analyze the cause of encephalopathy following short febrile illness in adults presenting to a tertiary care center in Northwestern part of India.

**Setting and Design:**

A prospective observational study carried out in a tertiary care center in the Northwestern India over a period of 1 year.

**Material and Methods:**

A total of 127 patients with fever of less than 2 weeks duration along with alteration in mentation were studied prospectively over a period of 12 months. The demographic variables were recorded in detail. In addition to routine investigations, cerebrospinal fluid analysis, noncontrast- and contrast-enhanced computed tomography, along with magnetic resonance imaging were performed in all the subjects.

**Statistical Analysis:**

The results were analyzed using SPSS statistical software. The values were expressed as mean with standard deviation for contiguous variable as percentage for the others.

**Results and Conclusion:**

Out of these, 70% had primary CNS infection as the etiology. A total of 33% patients had meningitis, 29.9% had evidence of meningoencephalitis, and 12.7% were diagnosed as sepsis-associated encephalopathy. These were followed by cerebral malaria, leptospirosis, and brain abscess as the cause of febrile encephalopathy in adults. Among the noninfectious causes, acute disseminated encephalomyelitis, cortical venous thrombosis, and neuroleptic malignant syndrome were documented in 2.36% each. In 11% of the patients, the final diagnosis could not be made in spite of the extensive investigations. Our study demonstrates that acute febrile encephalopathy in adults is a heterogeneous syndrome with primary CNS infections being the commonest etiology.

## INTRODUCTION

Acute onset fever with altered mentation is a problem commonly encountered by the physician in the emergency. “Acute febrile encephalopathy” is a term commonly used to identify this condition in which altered mental status either accompanies or follows a short febrile illness. It is a common condition leading to hospital admissions in both adults and children in India. Central nervous system (CNS) infections are the most common cause of nontraumatic coma.[1] The etiologic agent may be a virus, bacterium, or a parasite.[2] There is no systematic study available to define the etiology and the extent of this problem in adults.

The profile of “acute febrile encephalopathy” varies across different geographic regions and in different seasons in the same area. In India, we are faced with a dual problem, we have our age-old enemies, such as malaria, typhoid, and Japanese encephalitis (JE), and newer viruses causing this syndrome are also being increasingly recognized.[2]

The physician is faced with this challenge in the emergency of identification of the clinical syndrome, establishing the etiology and its prompt treatment not only to ensure survival but also to prevent long-term sequel in these patients. One has to be very meticulous while approaching such a patient because of the diversity of causes and equally large number of mimicking conditions. In untreated cases, mortality is very high and the survivors are often left with disabling neurologic sequelae.[3] Many a times even a detailed diagnostic workup may not identify a specific organism.[4] Nevertheless a detailed examination and workup is warranted as many conditions, such as herpes simplex encephalitis (HSE) and cerebral malaria are eminently treatable.[5]

Aim of this study was to understand the etiology of this syndrome affecting the adults aged more than 12 years, presenting to the medical emergency attached to the Nehru Hospital affiliated to Postgraduate Institute of Medical Education and Research at Chandigarh, in the Northwestern part of India.

## MATERIAL AND METHODS

This study was carried out in the medical emergency attached to the Nehru Hospital at Postgraduate Institute of Medical Education and Research, Chandigarh. Over a period of 12 months, we screened all the patients presenting to our emergency with fever and altered mentation and enrolled all consecutive adult patients who presented with fever of less than 15 days duration with altered mentation, either at onset or following fever, and lasting at least 24 h. We included all patients older than 12 years as our pediatrics emergency does not entertain any one older than 12 years. As we were trying to look at the profile of the patients presenting with short febrile illness and altered mentation, an effort was made to enroll all consecutive patients, there were no controls selected.

We excluded patients in whom the persistent alteration in mentation could be attributed to one or more deranged metabolic parameters, such as hypoglycemia < 50 mg/dL, hypoxia with partial pressure of oxygen < 60 mmHg, hypercarbia (PaCO_2_ ) > 50 mmHg, hyponatremia < 120 mg/dL, hypernatremia > 150 mg/dL, or azotemia (serum creatinine) > 3 mg/dL. Patients having cerebrovascular accident followed by fever were also excluded as structural lesion in the brain could be a reason for alteration in mentation.

The detailed history of the patients was recorded and the patients then underwent a detailed clinical examination. Hemogram, metabolic profile, chest radiography, and electrocardiogram were done in all patients. Peripheral smear for malarial parasite was examined in all the patients. A Histidine-rich protein-based immunochromatographic card test for *falciparum* malaria was performed in patients with negative peripheral smears where clinical suspicion for complicated malaria was high. Samples for blood cultures and urine cultures were collected and any clinically obvious site of sepsis was investigated. Lumbar puncture was carried out in all the patients at admission and cerebrospinal fluid (CSF) was analyzed for cytology, protein levels, CSF glucose to blood glucose ratio, gram stain, culture sensitivity, and CSF adenosine diaminase levels. All patients except 2, underwent noncontrast- and contrast-enhanced computed tomography (CT) of the brain. This was followed by a magnetic resonance imaging (MRI) scan of the brain using contrast. Two patients were directly taken for MRI brain scan as clinical suspicion of viral encephalitis was high. Serology for HSV and JE was performed but polymerase chain reaction was not standardized at the institute at the time of the study. The patients were classified into broad groups of meningitis, meningoencephalitis, and other clinical syndromes on the basis of predesignated diagnostic criteria [Table 1].

**Table 1:** Diagnostic criterion for specific diseases

Pyogenic meningitis	Fever with altered sensorium (without focal symptoms/signs) ± neck signs + CSF cytology (predominantly polymorphs) + meningeal enhancement on either CT or MRI scan
Meningoencephalitis	Fever with altered sensorium (with focal symptoms/signs) ± neck signs + CSF cytology (predominantly lymphocytes) + EEG/MRI/CT evidence of parenchymal disease
ADEM	Fever with altered sensorium (with focal symptoms/signs) + compatible CSF (raised CSF protein + normal CSF sugar + normal CSF cytology + diffuse white matter changes in the MRI
TBM	Fever with altered sensorium (with or without focal symptoms/signs) + CSF compatible with chronic meningitis + CSF ADA > 9/TB PCR positive
Cerebral malaria	Fever with altered sensorium (without focal symptoms/signs) with peripheral smear/HRP antigen test positive for malaria
Leptospirosis	Fever with altered sensorium (without focal symptoms/signs ± jaundice/renal dysfunction IgM ELISA for leptospira positive
Brain abscess	Fever with altered sensorium (with focal symptoms/signs) ± neck signs + CSF cytology (predominantly polymorphs) + Focal lesion on either CT or MRI scan
SAE	Underlying sepsis syndrome with normal CSF analysis, CT and MRI scan
CVT	Appropriate clinical setting+ fever with altered Sensorium (with focal symptoms/signs)+ evidence of CVT on MRI of the brain
NMS	Fever with altered sensorium (in the appropriate clinical setting) with normal CSF and imaging + raised total CPK
Heat stroke	Fever with altered sensorium (in the appropriate clinical setting) with normal CSF and imaging

ADA: adenosine diaminase; ADEM: acute disseminated encephalomyelitis; CPK: creatinine phosphokinase; CSF: cerebrospinal fluid; CT: computed tomography; CVT: cortical venous thrombosis; EEG: electroencephalogram; ELISA: enzyme-linked immunosorbent assay; HRP: histidine-rich protein; MRI: magnetic resonance imaging; NMS: neuroleptic malignant syndrome; PCR: polymerase chain reaction; SAE: sepsis-associated encephalopathy; TBM: tubercular meningitis

The data were analyzed using SPSS statistical software. The values were expressed as mean with standard deviation and percentages.

## RESULTS

**Figure 1:**
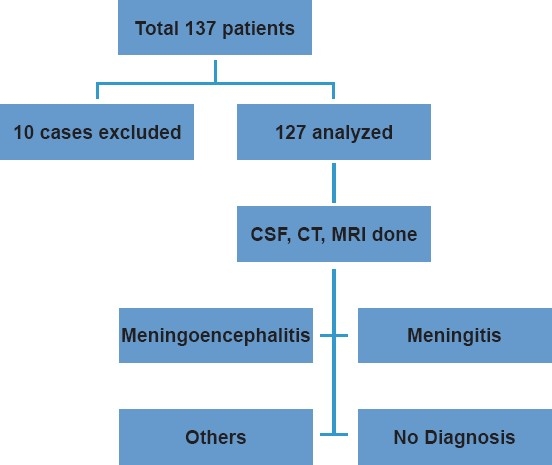
Evaluation of adult patients with acute febrile encephalopathy

During the study period, a total of 145 patients were admitted with complaints of fever and altered mentation to our emergency, of whom 137 patients had fever of less than 2 weeks duration along with the development of alteration in mentation after the onset of fever. Ten patients were excluded as the complete information was not available [Figure 1]. There was a male preponderance with 78.1% patients being male in our study group. The mean age was 30.14±14.79 years. The mean temperature was 38.4±0.8F at presentation. The mean pulse rate was 98±10.4 beats/min and mean systolic blood pressure was 112±16.9 mmHg. Fever and headache were the most common symptoms seen in 100% and 94% patients, respectively. Twenty-six percent of the patients had seizures at presentation. Neck stiffness was present in 26% patients. Of the total 127 patients, 70% had primary CNS infection as the etiology of febrile encephalopathy. A total of 42 patients had meningitis with 32 patients (25.2%) having acute pyogenic meningitis and 10 patients (7.87%) having tubercular meningitis (TBM). Thirty-eight patients (29.9%) had evidence of meningoencephalitis, which was followed by cerebral malaria, leptospirosis, and brain abscess as a cause of primary CNS infection. Sixteen patients (12.7%) had infections elsewhere and were diagnosed as sepsis-associated encephalopathy (SAE). Four patients (3.15%) were diagnosed as having cerebral malaria and 3 patients each (2.36%) had leptospirosis and acute disseminated encephalomyelitis. Three patients (2.36%) had cortical venous thrombosis (CVT), one secondary to ottitis media, and 2 patients each had brain abscess and neuroleptic malignant syndrome (NMS). In 1 patient, diagnosis of heat stroke was made. In 14 patients (11%), the final diagnosis could not be made in spite of the extensive investigations [Table 2].

**Table 2:** Etiology of acute febrile encephalopathy

Diagnosis	N = 127	%
Meningitis	42	33
Meningoencephalitis	38	29.9
Others	44	34.6
Unknown	14	11

A maximum number of patients (90/127) were seen during the period between May and October. This is the period of the year when the temperatures rise due to heat (May–July) followed by rains and postmonsoon period (July–October).

When further analyzed, of the total 38 patients with acute meningoencephalitis, a definitive diagnosis of viral meningoencephalitis attributable to HSV was possible only in 10 cases. This was based on the typical changes seen on MRI coupled with positive serology. A diagnosis of possible JE was made in 8 of 38 cases based on MRI findings and serology. Because we did not have the facilities for performing a complete viral panel, a presumptive diagnosis of viral meningoencephalitis was made in the remaining patients.

In patients with acute pyogenic meningitis, a diagnosis of pneumococcal meningitis was made in 6 patients and meningococcal meningitis in 2 patients based on the positive serology and gram stain. In the others, neither the gram stain nor cultures were able to point out specific causative agent. The reason for this could be the fact that we had hardly any treatment-naïve patients presenting to us at the center.

A majority of our patients were hemodynamically stable at presentation. The maximum number of patients presenting in shock were patients with SAE where 50% patients presented with blood pressure of <90 mmHg (9/18), 3/38 patients with meningoencephalitis presented in shock (18.42%), and 2 patients each with cerebral malaria and leptospirosis presented with shock and developed acute renal failure during the course of illness. None with acute pyogenic meningitis, NMS, or CVT were hemodynamically unstable. Seven of 14 (50%) patients in whom no definitive diagnosis could be established were in shock.

The patients were followed-up till discharge or death and the majority (83%) had a good outcome. The duration of hospital stay varied from 7 days (in majority) to up to 5 weeks. Only 20 patients had a hospital stay longer than 7 days and 7 patients had a prolonged stay (more than 4 weeks) due to persistence of neurologic sequel. All the patients with persistence of neurologic deficit belonged to meningoencephalitis group.

Of the total 127 patients, there were 21 deaths (16.5%). The maximum mortality was seen in patients with SAE with as many as 33% patients dying (6/18). Of the total number of 38 patients with meningoencephalitis, 7 succumbed to their illness (18.42%). One patient each died due to pyogenic meningitis and cerebral malaria and leptospirosis. Five patients out of 14 (35.7%) in whom no definitive diagnosis could be established succumbed to their illness. Presence of shock was one of the important clinical parameter determining the adverse outcome in these patients.

## DISCUSSION

Fever with altered mentation, is a common symptom complex leading to hospital admissions in both adults and children in our country and is also known as acute febrile encephalopathy. Fever with altered mental status is commonly produced by bacterial meningitis, Japanese B encephalitis, cerebral malarial, typhoid encephalopathy, and fulminant hepatic failure due to viral hepatitis.[2] Various studies in children with nontraumatic coma have shown that CNS infections are the commonest cause of nontraumatic coma.[1] However, no such studies are available in adults from our country. In the present study, we have tried to evaluate the common etiologies of acute febrile encephalopathy encountered in adults in a tertiary care center.

A study of nontraumatic coma in children has indicated that TBM, pyogenic meningitis, and encephalitis together constitute more than 90% of the cases.[1] In another study of 151 children, viral encephalitis was the most common etiology seen in 57 patients. A diagnosis other than viral encephalitis was reached in 94 patients (62.3%). Pyogenic meningitis was the most frequent diagnosis (33.8%) followed by TBM (7.9%), and cerebral malaria (5.2%) in the patient group of nonviral causes.[6] Our findings in adults are very similar to this study. Seventy percent of our patients had primary CNS infections as the major cause of altered mentation in febrile patients. This was followed by SAE as the second most common cause [Table 3] with 33% of our patients having meningitis, primary meningeal infection became the predominant cause of acute febrile encephalopathy in our study group. Meningoencephalitis of possible viral etiology was the second major group. The reason for the lower prevalence of TBM as an etiology for acute febrile encephalopathy in adults as compared with children could be the subacute/chronic presentation of TBM in adults.

**Table 3:** Details of the other cases

N = 34	Diagnosis	% (out of 127 cases)
Septic encephalopathy	16	12.6
Leptospira	4	3.14
ADEM	4	3.14
Cerebral malaria	4	3.14
CVT	3	2.36
NMS	2	1.57
Brain abscess	2	1.57
Heat stroke	1	0.78

ADEM: ACUTE DISSEMINATED ENCEPHALOMYELITIS; CVT: CORTICAL VENOUS THROMBOSIS; NMS: NEUROLEPTIC MALIGNANT SYNDROME

The important noninfectious etiologies that were identified included acute disseminated encephalomyelitis, CVT, and NMS, In as many as 14 of the 127 (11.76%) cases, even after all the investigations (CSF, CT, and MRI), we could not arrive at a definite diagnosis. This figure is much lower than that reported in the literature.[1,6]

We noted a male predominance in our study with males constituting around 60% of the study population and a 2:1 male:female ratio in patients with meningoencephalitis and a very similar male predominance was noted in a study in HSV encephalitis by Panagaria et al.[7] Although none of the CNS infections are known to have a male predominance, yet this apparent male predominance can be attributed to the male dominated social system where a sick male gets preferential medical attention.

It is postulated that alteration in sensorium in a patient with CNS infection indicates an element of parenchymal involvement.[6,8] This can explain the altered mentation in meningoencephalitis patients. In cerebral malaria, leptospirosis, and brain abscess, primary parenchymal involvement may be responsible for encephalopathy, but altered mentation in primary meningeal involvement is difficult to explain. Raised intracranial pressure may contribute to altered mentation to some extent. The reason for altered sensorium in meningitis is postulated to be the spillage of inflammatory cells to the adjacent brain parenchyma and the resultant parenchymal involvement.[8] In NMS and SAE, metabolic alterations and inflammatory cytokines may play an important role in the pathogenesis of encephalopathy rather than direct parenchymal involvement. Encephalopathy in CVT may be a direct result of the parenchymal involvement, but the fever may be due to the predisposing condition.

HSV is a common cause of sporadic encephalitis around the world.[5] Postmonsoon JE has been reported from many parts of India. The less common varicella encephalitis tends to be fatal in immunocompromised patients. Among the other identifiable viruses, enterovirus, JE virus, and mumps are important agents.[6] In our study, the most commonly identifiable cause of encephalitis was herpes simplex encephalitis followed by JE. The complete virologic screen was not available to us and hence, we could not identify the culprit virus in a substantial number of our patients.

Cerebral malaria, the potentially fatal complication of falciparum malaria is an important cause of unarousable coma in febrile patients in endemic areas. In the endemic areas, cerebral malaria remains an important differential in patients presenting with acute fever and altered sensorium.[2] Postmonsoon surge in malaria cases coincides with that of viral encephalitis and the common symptomatology may be confusing to the physicians. Because Northwestern India is not endemic for falciparum malaria, we did not encounter a large number of cerebral malaria cases in our study group; however, it is a fairly common cause of fever in the adjoining state of Rajasthan.

Dengue hemorrhagic fever presents as a short febrile illness and thrombocytopenia but may rarely present with alteration in sensorium. In a study, 62 of 265 patients with acute febrile encephalopathy from central India tested positive for dengue serology but only 39 met the criterion for definite dengue virus infection.[9] Although we do see a lot of dengue virus infections every year, in the present study, we did not encounter any patient with dengue fever presenting as short febrile illness with altered mentation.

SAE is a poorly understood CNS condition that is associated with a wide range of manifestations from lethargy to overt delirium in sepsis patients. SAE has serious prognostic implications and it is an important predictor of higher 6 months mortality.[10] Since a large number of patients present to emergency in a tertiary care hospital with sepsis, it becomes an important differential diagnosis of acute febrile encephalopathy in adults. In our study population, we did encounter SAE as an important cause of acute febrile encephalopathy in adults.

NMS, which arises as a complication of antipsychotic therapy or in the setting of abrupt withdrawal of levodopa, can present as febrile encephalopathy. In our study group, this formed an important cause of febrile encephalopathy. Drugs, such as dantrolene have also been implicated as a cause of acute febrile encephalopathy in adults but are difficult to implicate.[11] In the absence of any clue on diagnostic evaluation, a good clinical history and raised levels of creatinine phosphokinase enzyme can help in clinching this diagnosis. The prompt recognition of this condition is extremely important as the treatment is radically different and life saving.

Many acutely ill febrile patients with encephalopathy may make complete recovery once the underlying cause is treated but considerable skill is required to correctly diagnose the underlying etiology. The majority of our patients did make a complete recovery; however, a significant number of patients (16.8%) died and a small number (5.5%) were left with neurologic sequel. Delayed neurologic recovery and sequel are well described with meningoencephalitis.[2,3] The fact that maximum mortality was seen in patients with SAE signifies that multiorgan dysfunction may have contributed to a large extent in these patients. Raised intracranial pressure may have contributed to mortality in patients with meningoencephalitis. Mortality was also high in patients in whom a definitive diagnosis could not be made.

## CONCLUSIONS

Acute febrile encephalopathy is a heterogenous syndrome. Many causative agents can result in a very similar kind of presentation. In our study group, CNS infections, particularly meningoencephalitis, acute pyogenic meningitis, and other CNS infections constituted a major chunk. Unlike children, TBM was not a very major cause of acute febrile encephalopathy in adults.
